# Essential factors involved in the precise targeting and insertion of telomere-specific non-LTR retrotransposon, SART1Bm

**DOI:** 10.1038/s41598-020-65925-x

**Published:** 2020-06-02

**Authors:** Narisu Nichuguti, Haruhiko Fujiwara

**Affiliations:** Department of Integrated Biosciences, Graduate School of Frontier Sciences, The University of Tokyo, Kashiwa, Chiba 277-8562 Japan

**Keywords:** DNA transposable elements, Transposition

## Abstract

Telomere length maintenance is essential for most eukaryotes to ensure genome stability and integrity. A non-long terminal repeat (LTR) retrotransposon, SART1Bm, targets telomeric repeats (TTAGG)n of the silkworm *Bombyx mori* and is presumably involved in telomere length maintenance. However, how many telomeric repeats are required for its retrotransposition and how reverse transcription is initiated at the target site are not well understood. Here, using an *ex vivo* and *trans-in vivo* recombinant baculovirus retrotransposition system, we demonstrated that SART1Bm requires at least three (TTAGG) telomeric repeats and a longer poly(A) tail for its accurate retrotransposition. We found that SART1Bm retrotransposed only in the third (TTAGG) tract of three repeats and that the A residue of the (TTAGG) unit was essential for its retrotransposition. Interestingly, SART1Bm also retrotransposed into telomeric repeats of other species, such as human (TTAGGG)n repeats, albeit with low retrotransposition efficiency. We further showed that the reverse transcription of SART1Bm occurred inaccurately at the internal site of the 3′ untranslated region (UTR) when using a short poly(A) tail but at the accurate site when using a longer poly(A) tail. These findings promote our understanding of the general mechanisms of site-specific retrotransposition and aid the development of a site-specific gene knock-in tool.

## Introduction

Telomere length maintenance is important for genome protection, replication, aging and disease^1^. However, telomere shortens in every cell replication causing the “end-replication problem”^2,3^. For the elongation or maintenance of telomeres, most eukaryotes use telomerase to add DNA repeats^4,5^. Alternatively, some organisms have evolved non-canonical telomere elongation mechanism such as recombination, and retrotransposon based telomere elongation^6,7^.

Although most insects retain telomeric repeats and telomerase, some have lost telomeric repeats, which is compensated by non-long terminal repeat (LTR) retrotransposon^8–10^. For instance, *Drosophila melanogaster* harbors no telomeric repeats and completely lacks telomerase activity^11,12^. Instead, telomere-specialized non-LTR retrotransposons such as HeT-A, TART, and TAHRE retrotranspose into the end of chromosomes to preserve distal and unique telomere sequences^6,13^.

In contrast, in the *Bombyx mori* genome, there are not only (TTAGG/CCTAA)n telomeric repeats but also telomere-specific non-LTR retrotransposon families, SART and TRAS, which insert into telomeric repeats in different orientations^14,15^. TRAS is inserted specifically into the site between the C and T of the (CCTAA)n strand (bottom strand), and SART is inserted specifically between the T and A of the (TTAGG)n strand (top strand)^7,14,16^. In *B. mori*, studies have revealed that a telomerase activity is undetectable by the telomeric repeat amplification protocol (TRAP)^17^ and that the telomere reverse transcriptase (TERT) has an N-terminal deficient structure and a lower transcriptional and translational activity^18^. In the red flour beetle *Tribolium castaneum*, it has also been shown that there are similar structures and features of telomeric-repeats, telomere-specific retrotransposons, and TERT genes^18^. Thus, we hypothesize that telomere-specific non-LTR retrotransposons contribute to telomere length maintenance of these insects.

**Figure 1:**
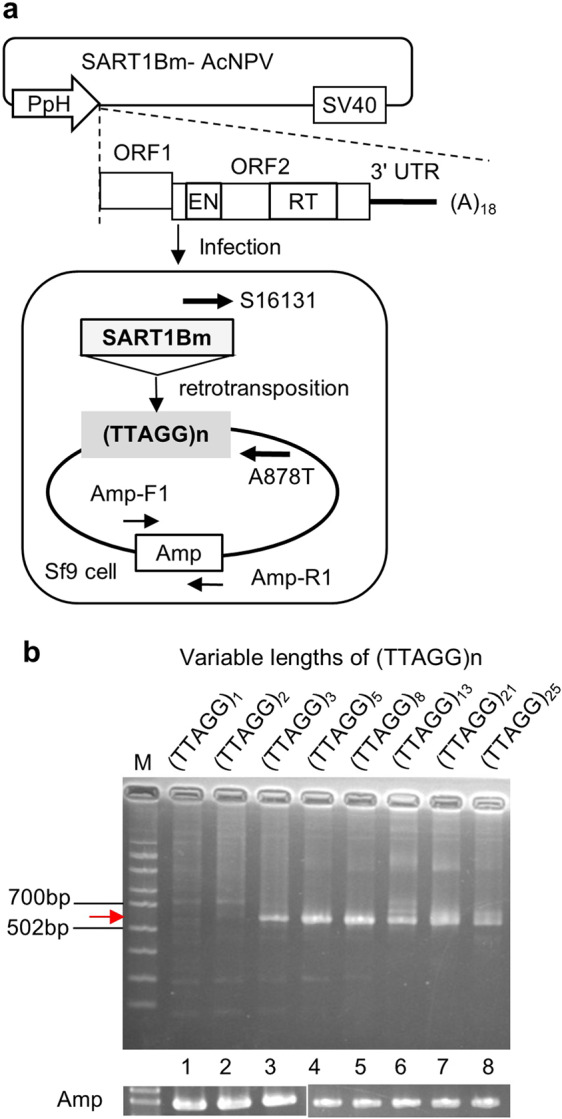
*Ex vivo* retrotransposition assay for SART1Bm with variable lengths of target site. (**a**) Schematic overview of the SART1Bm *ex vivo* retrotransposition assay. Target of (TTAGG)n-plasmids (gray box) are transfected into Sf9 cells. Then, baculovirus containing SART1Bm (SART1Bm- AcNPV) is infected into Sf9 cells. Retrotransposition to the target plasmids is detected by a SART1Bm primer (S16131) and a primer designed in the common region of the target plasmids (A878T), indicated by arrows. (**b**) PCR results of the *ex vivo* retrotransposition of SART1Bm into the target plasmids with variable lengths of TTAGG repeats. The smeared expected PCR bands in lanes three to eight lanes represent the retrotransposition event (572 bp + telomeric repeats) indicated by red arrowhead. Amp: The ampicillin resistance gene was used as an internal control. Full-length gels are presented in Supplementary Figure S1. PpH, polyhedrin promoter; ORF, open reading frame; (A)18, polyA 18 nucleotide tail; SV40, SV40 PolyA signal.

A major element in the silkworm SART family (SART1Bm) is 6.7 kb in length and is inserted specifically between the T and A of the TTAGG strand^16^. SART1Bm starts from 5′ untranslated region (UTR), encodes two open reading frame (ORF) proteins (ORF1p and ORF2p), and ends with 3’ UTR followed by a poly(A) tail (Fig. 1a). ORF1 encodes an RNA binding domain^19^, while ORF2 encodes reverse transcriptase (RT) and apurinic/apyrimidinic(AP)-like endonuclease (EN) domains, which are essential for the SART1Bm retrotransposition^20^. As SART1Bm has high transcriptional activity and specificity for integration^21^, it has been used as a good model to study the site-specific retrotransposition mechanism.

Our group previously established a baculovirus-based *in vivo* retrotransposition assay involving the expression of SART1Bm under control of the polyhedrin promoter (PpH) from *Autographa californica* nuclear polyhedrosis virus (AcNPV) in *Spodoptera frugiperda* 9 (Sf9) cells. The retrotransposition of SART1Bm elements into target sites of the Sf9 cell genome can be easily detected by polymerase chain reaction (PCR), which shows the sequence-specific retrotransposition of SART1Bm *in vivo*^20^. Swapping the EN domain of SART1Bm with that of TRAS1Bm can also change the sequence specificity for integration from SART1Bm to TRAS1Bm^20^. In addition, TRAS1Bm EN domain that cuts telomere repeats was characterized by an *in vitro* study^22^, suggesting the involvement in target-site selection. Although target-site recognition is the first step in the integration reaction and is believed to be achieved primarily through the specificity of the EN domain, there is still a lack of a comprehensive understanding of the target-site recognition and the sequence specificity of site-specific non-LTR elements.

To investigate the target-site recognition mechanism in SART1Bm, we here used an *ex vivo* retrotransposition assay (Fig. 1a)^23,24^. Within this system, we first transfected a plasmid bearing the exogenous target sequence into Sf9 cells and thereafter infected a SART1Bm recombinant baculovirus, enabling easy assaying of target sequence mutants simply by constructing the plasmid. Via this approach, we showed that at least three (TTAGG) repeats and the nucleotide A of the (TTAGG) repeats are essential for the SART1Bm retrotransposition.

Non-LTR retrotransposons are usually reverse transcribed from a specific sequence of their template mRNA. However, the mechanism underlying the accurate reverse transcription is still unclear. Notably, most non-LTR retrotransposons including R1-clade elements end with a poly(A) tail, which has been suggested to be involved in this mechanism. Previously, we showed that the rDNA-specific R1 element R7Ag requires a longer poly(A) tract of 20 oligo(A) for the accurate initiation of reverse transcription^24^. In this study, to test whether a longer poly(A) tail is required for a telomere-specific R1 element SART1Bm, we generated several SART1Bm constructs with variable lengths of poly(A) tract and conducted a *trans-in vivo* retrotransposition assay, a novel approach established in this study. Our results revealed that the deletion or reduction of the poly(A) tract increased the rate of inaccurate reverse transcription in the internal site of 3′ UTR, but longer poly(A) tails recovered the accurate reverse transcription. Although there are major differences between the target and 3′ UTR sequences of R7Ag and SART1Bm, the ribonucleoprotein (RNP) particle recognizes a long poly (A) tail and initiates reverse transcription from the accurate site, the 3′-end of 3′ UTR.

## Results

### SART1Bm requires at least three repeats of the (TTAGG) target site for retrotransposition

**Figure 2:**
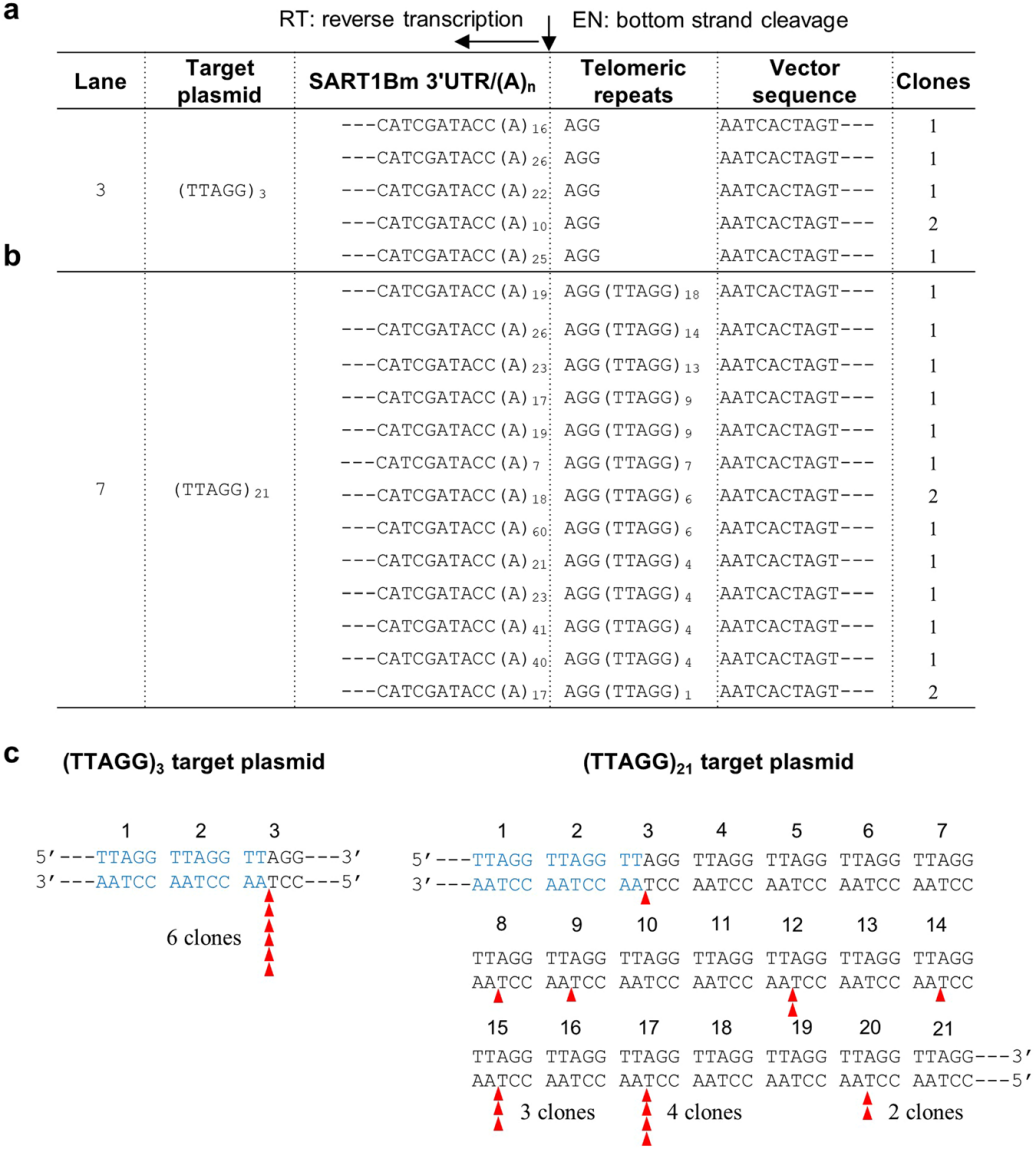
Insertion sites of SART1Bm in telomeric repeats of target plasmid in *ex vivo* retrotransposition. (**a**) 3′-junction clones obtained from the PCR product for the (TTAGG)_3_ target (Fig. 1a, lane 3). (**b**) 3′-junction clones obtained from the PCR product for the (TTAGG)_21_ target (Figure 1a, lane 7). The number of PCR lanes and the target plasmid in Fig. 1a are shown on the left. The 3′ junction sequences, which are the boundaries between the inserted retrotransposon copies (SART1Bm 3′ UTR) and the host target sites (telomeric repeats), are shown in each line. The vector sequence indicates the sequence from the target plasmid. The clone number of each type is shown on the right. The vertical arrowhead indicates the endonuclease (EN) bottom strand cleavage site. The horizontal arrowhead indicates the reverse transcription start site designated as nucleotide position 0. (**c**) Loci of SART1Bm insertion into (TTAGG) repeats in the target plasmid. Each telomeric tract is numbered from the 5′ side. Red arrowheads show the positions and numbers of clones inserted into the locus. Blue sequences in the target plasmid indicate the telomeric sequences that contain no SART1Bm insertions.

Using *ex vivo* assay, we first investigated how SART1Bm recognizes the target site of telomeric repeats (TTAGG)_n_. We constructed target plasmids with variable lengths of the (TTAGG) tract and transfected them into Sf9 cells. After infection of the cells with the SART1Bm baculovirus, retrotransposition of SART1Bm was detected by PCR amplification of the 3′-junction of the retrotransposed SART1Bm and telomeric repeats in the plasmid (Fig. 1a). Although PCR bands were not revealed for one or two (TTAGG) telomeric repeats as target (Fig. 1b, lanes 1 and 2), 3 to 25 (TTAGG) telomeric repeats yielded PCR bands (between the marker of 502 and 700 bp; 572 bp + telomeric repeats), which represent the exact retrotransposition event of SART1Bm (Fig. 1b, lanes 3–8). These results indicate that at least three (TTAGG) repeats are required for the SART1Bm retrotransposition (Fig. 1b, lane 3). From (TTAGG)_3_ and (TTAGG)_21_ target samples, we further cloned the PCR bands, sequenced 16 clones each, and characterized transposed clones (Fig. 1b, lanes 3 and 7). Of these 16 sequenced clones, six showed the accurate retrotransposition into (TTAGG)_3_ and 15 showed accurate retrotransposition into (TTAGG)_21_ (Fig. 2a, b). The remaining clones were from the genomic sequences of baculovirus. The clones from genomic sequences are not nonspecific insertions but just part of the baculovirus vector sequence. We observed four types of baculovirus sequence, which arise as PCR artifacts with a mismatched primer annealed to the baculovirus genome (Supplementary Table 2, types 1 to 4). The ampicillin gene of the target plasmid was used as an internal control and detected by a primer designed in the ampicillin sequence (Amp-F1 and Amp-R1) (Fig. 1a, b; Supplementary Fig. S1). All retrotransposed clones showed the poly(A) tail joined to the AGG sequence of telomeric repeats in the 3′-junction region, suggesting that first the EN domain-mediated bottom-strand cleavage of the target telomere occurred between the TT and AGG of the (TTAGG) repeats; second, an accurate reverse transcription of mRNA occurred from the 3′ UTR end of the variable length poly(A) tail (Fig. 2a,b). This structure is identical to the 3′-junction sequence of SART1Bm observed in the endogenous *Bombyx* genome^16^. Based on the sequencing results, we summarized the SART1Bm insertion loci in (TTAGG) repeats (Fig. 2c). In (TTAGG)_3_ telomeric repeats of the target plasmid, all retrotransposed clones (six clones) were inserted in the third (TTAGG) tract but not into the first or second tract (Fig. 2c, left panel). Moreover, in (TTAGG)_21_ repeats, insertions frequently occurred in the 15^th^ and 17^th^ tracts but not in the 1st and 2nd tracts (Fig. 2c, right panel). These results suggest that the upstream 12 bp sequence (3′- AATCCAATCCAA↑-5′; ↑ denotes the nicking site) in the bottom strand or three telomeric repeats is important for the recognition of SART1Bm for insertion into the target site.

### Nucleotide A in (TTAGG) repeats is essential for SART1Bm retrotransposition

**Figure 3:**
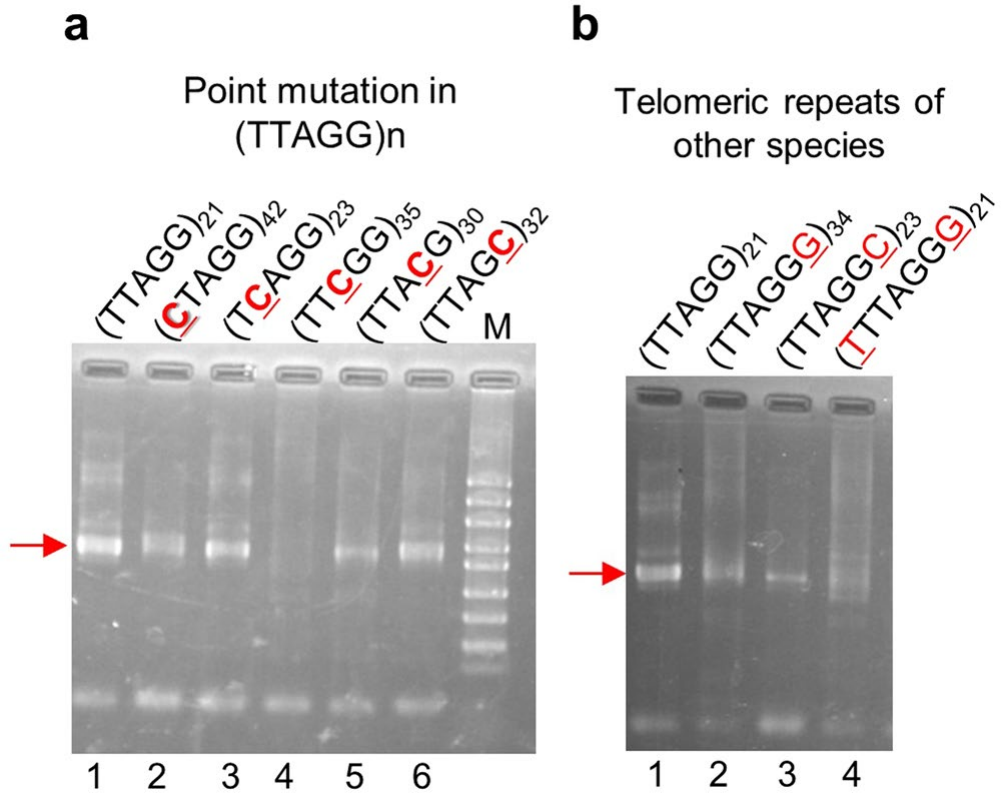
*Ex vivo* retrotransposition assay for SART1Bm with point mutated (TTAGG) repeats and telomere repeats of other species. (**a**) The 3′-junction PCR analysis of *ex vivo* retrotransposition with point mutated (TTAGG) repeats. Each nucleotide of the TTAGG unit is mutated individually to a cytosine. Mutated nucleotides are red and underlined. (**b**) PCR results of the *ex vivo* retrotransposition of SART1Bm into the target plasmids with telomere repeats of other species. *H. sapiens* (TTAGGG)_34_, *C. elegans* (TTAGGC)_23_ and *A. thaliana* (TTTAGGG)_21_. The red arrowhead indicates the expected PCR band.

To further determine the involvement of each nucleotide in (TTAGG) repeats for SART1Bm retrotransposition, we generated five mutated target plasmids, each with a single nucleotide substitution (N to C replacement) on each (TTAGG) repeat of the target plasmid. Briefly, we altered one nucleotide of the (TTAGG) units to a cytosine (C) residue, generating five different target-site plasmids: (CTAGG)_42_, (TCAGG)_23_, (TTCGG)_35_, (TTACG)_30_, and (TTAGC)_32_ (mutated nucleotides are underlined) (Fig. 3a). We then performed an *ex vivo* retrotransposition assay using these mutants as targets. In four mutants ([CTAGG]_42_, [TCAGG]_23_, [TTACG]_30_ and [TTAGC)_32_]), we observed the PCR band that represents the SART1Bm retrotransposition as similar to the WT target (Fig. 3a). However, a mutation of the third adenine to cytosine ([TTCGG]_35_) abolished SART1Bm’s retrotransposition (Fig. 3a, lane 4). This result suggests that the nucleotide A in the (TTAGG) repeats is essential for the SART1Bm retrotransposition. We cloned and sequenced PCR products of 16 clones for each construct. Sequencing analysis revealed that SART1Bm retrotransposed into the accurate site in four target mutants, although no retrotransposed clone was obtained in the mutant (TTCGG)_35_ (Supplementary Table 3). The remaining clones originated from genomic sequences of baculovirus (Supplementary Table 2, types 5–8). We considered that nucleotide A of the (TTAGG) (specifically nucleotide T in the bottom strand) unit is important for the accurate cleavage by SART1Bm EN or the target recognition by SART1Bm RNP. Notably, consistent with a previous study^23^, SART1Bm could insert into the point mutant (TCAGG)_23_ (Fig. 3a, lane 3), corresponding to the telomeric repeats of the red flour beetle *T. castaneum*. This result raised a question of whether SART1Bm can recognize and cut the telomeric repeats of other organisms.

### SART1Bm retrotransposes into telomeric repeats of other species

We next examined whether SART1Bm can retrotranspose into telomeric repeats of species other than *B. mori*. By adding nucleotides onto each telomeric (TTAGG) repeat, we constructed telomeric repeats of *Homo sapiens* (TTAGGG)_34_, *Caenorhabditis elegans* (TTAGGC)_23_, and *Arabidopsis thaliana* (TTTAGGG)_21_ in target plasmids (added nucleotides are underlined). Using (TTAGGG)_34_ or (TTAGGC)_23_ as a target, SART1Bm showed PCR bands with a signal intensity that was lower than those of the host target (TTAGG)_21_ (Fig. 3b, lanes 1–3). When (TTTAGGG)_21_ was used as a target, SART1Bm showed a weak, smeared band (Fig. 3b, lane 4). Sequencing analysis confirmed that these bands represented retrotransposition events (Supplementary Table 4). Among 16 sequenced clones for each target, one or two was inserted accurately into the (TT↑AGG) site of these telomeric repeats (Supplementary Table 4). The remaining clones originated from genomic sequences of baculovirus (Supplementary Table 2, types 9–12). These results suggest that SART1Bm may retrotranspose into telomeric repeats of other species, albeit with low efficiency. In addition, we previously developed a novel system for delivering genes using baculovirus in *Bombyx mori* and the tussock moth *Orgyia recens*, in which SART1Bm integrates specifically into telomeric repeats of (TTAGG)n^25^. We here found that SART1Bm can retrotranspose into human telomeric repeats (TTAGGG)n, suggesting that it can be used as a genetic tool not only in insects^25^ but also in other organisms.

### Establishment of a novel *trans-in vivo* retrotransposition assay

**Figure 4:**
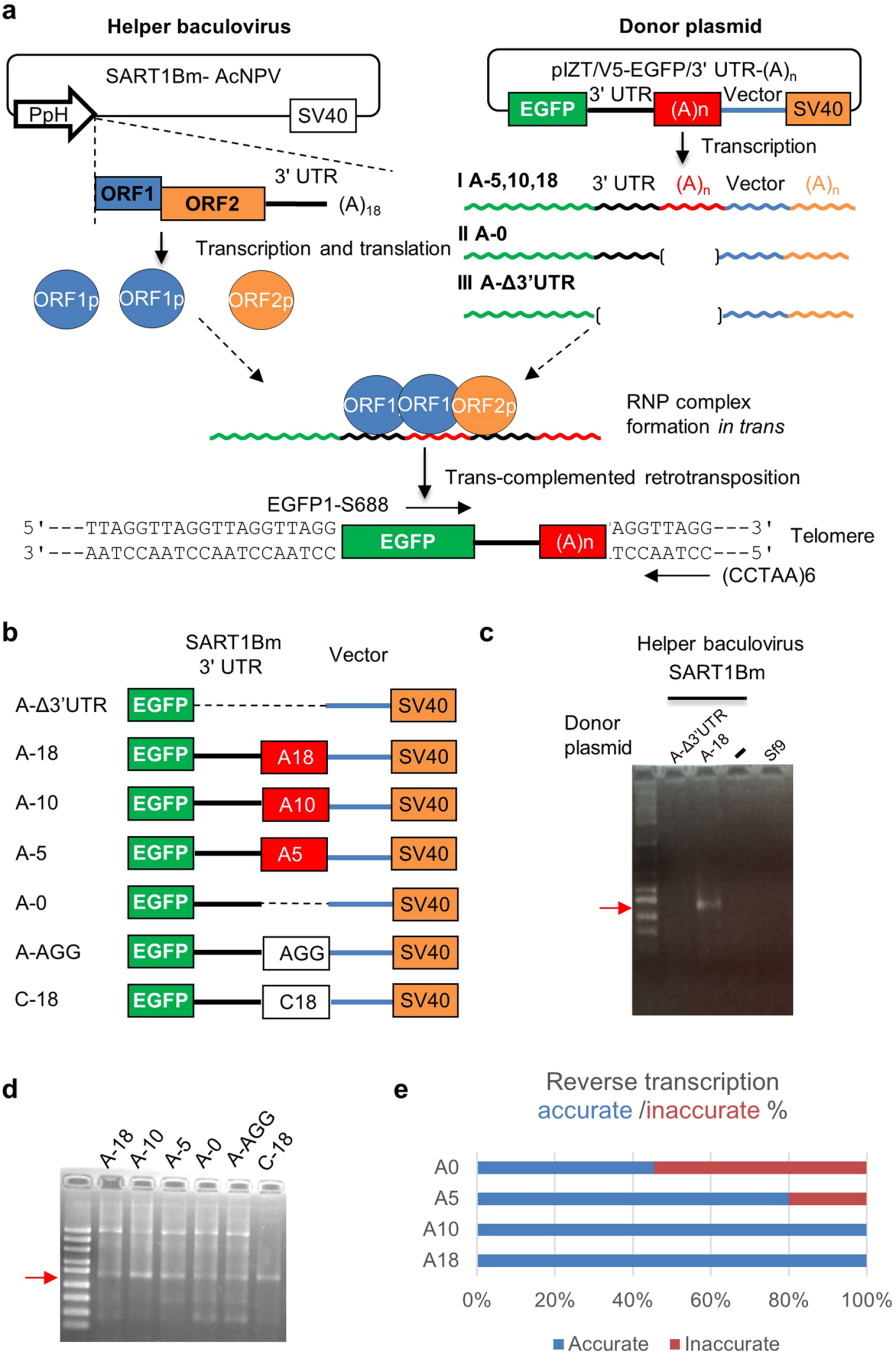
*Trans-in vivo* retrotransposition assay in SART1Bm. (**a**) “Helper” SART1Bm baculovirus (SART1Bm-AcNPV) expresses SART1Bm ORF1p (blue oval) and ORF2p (orange oval). “Donor” plasmids [pIZT/V5-EGFP/3’ UTR-(A)n plasmid] express mRNA of EGFP fused with the SART1Bm 3′ UTR end with variable lengths of poly(**A**). “Donor” plasmids contain the SV40 polyadenylation signal (SV40). After transfection of “donor” plasmids, the trans-complementation assay measures the ability of “helper” baculovirus to retrotranspose a “donor” EGFP/3’ UTR-(A)n RNA that contains a variable length of poly(A), which leads to the retrotransposition event. Type I has 3’ UTR with two types of poly(A) tail. Type II lacks an encoded poly(A) tail and type III lacks both 3’ UTR and the encoded poly(A) tail. Parentheses indicate the deleted region. The 3′ junction of retrotransposed copies was detected by PCR with primers EGFP1 S688 and (CCTAA)6 (black arrows). RNP indicates the ribonucleoprotein complex. (**b**) Construct A-Δ3’UTR lacks SART1Bm 3′ UTR and the poly(A) tail. Constructs A-18, A-10, and A-5 contain various lengths of poly(A) tail directly connected to the 3′ UTR end. Construct A-0 lacks a poly(A) tail. Constructs AGG and C-18 contain AGG sequences and poly(C)_18_ tail directly connected to the 3′ UTR end in place of poly (A) tail, respectively. Dotted line indicates the deleted region. (**c**) PCR results for the *trans-in vivo* retrotransposition assay. A-Δ3’UTR, EGFP transfected Sf9 cells with the SART1Bm infection; A-18, EGFP/3’ UTR-(A)_18_ transfected Sf9 cells with the SART1Bm infection; (−), Sf9 cells with the SART1Bm infection; Sf9, Sf9 cells without the SART1Bm infection. Red arrow indicates the successful *trans-in vivo* retrotransposition band with the expected size. Molecular sizes are shown on the left. (**d**) PCR results of *trans-in vivo* retrotransposition assay. Red arrow indicates the expected PCR band (614 bp + telomeric repeats). (**e**) Summary of the accurate/inaccurate reverse transcription rates in SART1Bm. When the poly(A) length increased, the rate of inaccurate reverse transcription decreased in both elements.

After recognizing and nicking the target site, SART1Bm is reverse transcribed from a specific sequence of its template mRNA using the target site as a primer. However, the mechanism underlying the accurate reverse transcription is still unclear. Previously, we showed that the lack of poly(A) resulted in inaccurate reverse transcription^26^. In addition, R7Ag requires a longer poly(A) tract of 20 oligo(A) for the accurate initiation of reverse transcription, which suggested that the poly(A) tail may be involved in this mechanism. To study the essential role of the poly(A) tail in the retrotransposition event, we developed a novel *trans*-*in vivo* retrotransposition assay for SART1Bm (Fig. 4a). Using a baculovirus-based *in vivo* retrotransposition assay Osanai *et al*. (2004), showed that SART1Bm proteins (helper) recognize their 3′ UTR *in trans* to retrotranspose enhanced green fluorescence protein (EGFP) mRNA fused with a SART1Bm 3′ UTR (donor) into telomeric repeats. We modified this *in vivo* retrotransposition assay, by exchanging the donor baculovirus to a plasmid with the pIZT/V5 backbone and tested whether mRNA transcribed from the donor plasmid can be inserted into telomeric repeats. Donor plasmids include the EGFP-encoding gene with SART1Bm 3′UTR and encode a poly(A) tail followed by a plasmid poly(A) signal. Donor plasmid can generate three types of transcript. Type I has 3′ UTR with two poly(A) tails. Type II lacks an encoded poly(A) tail and type III lacks both 3′ UTR and an encoded poly(A) tail (Fig. 4a). Only in the presence of 3′ UTR (types I and II), SART1Bm proteins (helper) recognize their 3′ UTR *in trans* to retrotranspose the EGFP mRNA fused with a SART1Bm 3′ UTR (donor) into telomeric repeats (Fig. 4a).This new method enables donor constructs to be made more easily than the former method of Osanai *et al*. (2004). We used a set of primers EGFP1 S688 and (CCTAA)6 designed for amplifying the 3′ junction region between the transposed EGFP sequence and telomeric repeats. If the helper (SART1Bm) and donor [EGFP1/3′ UTR-(A)_n_] can retrotranspose by *trans*-complementation, a 700 bp PCR band resulting from such retrotransposition is observed (Fig. 4a). We observed the PCR band only when both the helper and a donor EGFP with 3′ UTR of SART1Bm were introduced into Sf9 cells (Fig. 4b,c, lane A-18). To confirm that retrotransposition occurred accurately, the PCR products were cloned and sequenced. Our findings showed that all six clones were accurately inserted into the telomeric repeats and reverse transcribed precisely from the poly(A) tract at the end of the 3′ UTR of EGFP/3′ UTR-(A)_18_ (Supplementary Table 5). In contrast, SART1Bm (helper) did not cause the retrotransposition in Sf9 cells when EGFP without the 3′ UTR-(A)_18_ region was used (Fig. 4c, lane A-Δ3′-UTR). This indicates that the retrotransposition of EGFP/3′ UTR is mediated by SART1Bm ORF proteins provided *in trans* from the helper construct, which recognize the 3′ UTR of SART1Bm. As negative controls, helper SART1Bm alone (Fig. 4c, lane -) or Sf9 cell alone (Fig. 4c, lane Sf9) showed no PCR bands. We named this novel method the “*trans-in vivo* retrotransposition assay”.

### A long poly(A) tail at the end of the 3′ UTR is critical for accurate SART1Bm reverse transcription

Next, to clarify the role of the length of the poly(A) tail in SART1Bm retrotransposition, we generated a series of EGFP/3′ UTR plasmids with a variable-length poly(A) tract. As the average poly(A) tail length in genomic copies of SART1Bm is 20 bp, we generated constructs of A-0, lacking a poly(A) tail, as well as A-5, A-10 and A-18 with poly(A) tails of 5,10, and 18 nucleotide oligo(A) located immediately downstream of the 3′ UTR, respectively and conducted *trans-in vivo* retrotransposition assays (Fig. 4b). All constructs with shorter poly(A) tails showed the same PCR band near the 700 bp (Fig. 4d). The results of detailed sequence analyses of the transposed EGFP/3′ UTR and (TTAGG) junctions for each construct are shown in Supplementary Tables 6 and 7. Of the 11 junction products cloned from the A-0, we observed that five clones exhibited the accurate cleavage at TT and AGG; reverse transcription was initiated accurately from the 3′ UTR end of SART1Bm, in which sequences were followed with a variable length of the poly(A) tail which might be added by an unclear mechanism as discussed below (Supplementary Table 6). Interestingly, although five of the remaining six clones exhibited accurate cleavage, the reverse transcription was initiated at internal sites within the 3′ UTR, at −394 nucleotides upstream from the junction site (Supplementary Table 6, shown in red). This internal initiation site starts from the telomeric-repeat-like sequence (AGG), which may anneal to cleave the bottom-site TCC to initiate reverse transcription (as discussed later). In one clone with −398 internal initiation, the non-template nucleotide of T was found at the junction site between the 3′ UTR sequences and the (TTAGG) repeats (Supplementary Table 6). Similarly, of the 10 clones obtained from the A-5 construct, eight clones accurately initiated reverse transcription at the end of the poly(A) tail. However, two clones initiated at −394 nucleotides from the junction site, similar to the findings in the A-0 construct (Supplementary Table 6). In the A-10 and A-18 constructs, all clones initiated reverse transcription accurately from the poly(A) tail end (Supplementary Table 7). The rates of accurate/inaccurate reverse transcription events are summarized in Fig. 4e. These results showed that the A-0 construct with a lower rate of accurate initiation ration (36%) was not used appropriately as a template for accurate reverse transcription (Fig. 4e). The rate of accurate reverse transcription rate for the A-5 construct increased to 80%, and those for A-10 and A-18 constructs reached 100% (Fig. 4e). This indicated that a longer poly(A) tail in the donor construct is necessary for ORF proteins of SART1Bm to recognize and start reverse transcription accurately.

### Replacement of the poly(A) tail with another sequence does not abolish retrotransposition

A previous study showed that in the deletion construct of 296–461 for the 3′ UTR of SART1Bm, reverse transcription occurs mostly from several telomeric-repeat-like GGUU sequences just downstream of the second stem-loop within 3′ UTR^26^. It was hypothesized that short telomeric-repeat-like sequences within the 3′ UTR of SART1Bm mRNA anneal to the bottom strand of the (TTAGG)_n_ repeats in the target DNA. As shown above, the short AGG at position −394 in the 3′ UTR can potentially anneal with the target DNA during the initiation of reverse transcription. To determine the functional role of the (AGG) sequence, we next tested whether the construct A-AGG in which the poly(A) tail was replaced with the (AGG) sequence, would increase the efficiency or accuracy of SART1Bm retrotransposition compared with that of construct A-0, which lacks a poly(A) tail (Fig. 4b, A-AGG). We also established the C-18 construct to investigate whether another simple poly(C) repeat is also an efficient initiator of SART1Bm reverse transcription (Fig. 4b, C-18). In both constructs, we observed the PCR bands representing the retrotransposition (Fig. 4d, lanes A-AGG and C-18). Cloning and sequencing of the PCR bands in the A-AGG construct showed the presence of two types of clone: One was reverse transcribed from an internal site of the 3′ UTR (−394) and the other was accurately from the end of the poly(A) tract at the 3′ UTR in place of the AGG tail (Supplementary Table 8), although this poly (A) tail was not included in the original construct. Similarly, in most of the clones in the C-18 construct (eight of nine clones), reverse transcription occurred at the poly(A) tail after the 3′ UTR in place of the original poly(C) tail. This poly (A) tail was not included in the original construct. In the C-18 construct, one clone showed the internal AGG initiation within the 3′ UTR (−233) (Supplementary Table 8, shown in red). Intriguingly, in both constructs (AGG and C-18), poly(A) nucleotides might be added to the 3′ UTR end after transcription by some cellular factors or just before the start of target primed reverse transcription (TPRT) by the RT domain.

## Discussion

**Figure 5:**
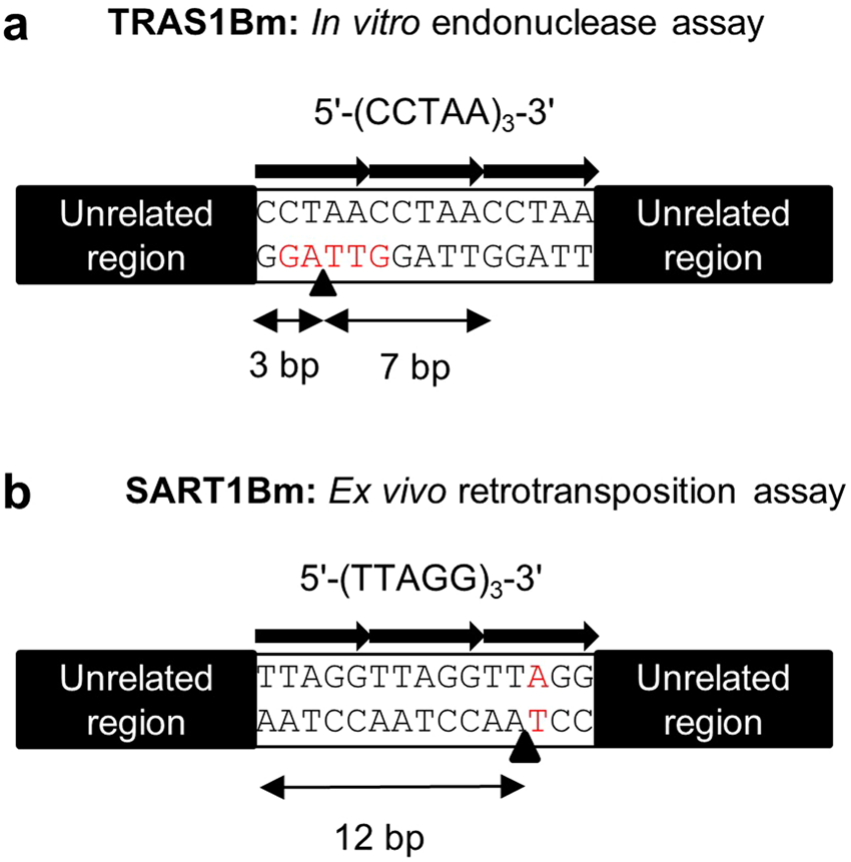
Comparison of target-site recognition mechanisms for TRAS1 and SART1.Telomere-specific non-LTR retrotransposons SART1Bm and TRAS1Bm show similar target-site recognition features. (**a**) The *in vitro* endonuclease assay showed that TRAS1Bm recognizes a 10 bp sequence around the nicking site. (**b**) The *ex vivo* assay showed that SART1Bm recognizes a shorter 12 bp sequence around the nicking site. In both cases, the flanking sequence (unrelated region) does not influence the EN cleavage activity.

The telomere-specific non-LTR retrotransposon TRAS1Bm and SART1Bm insert into the telomeric repeats of (TTAGG/CCTAA)n in opposite directions Anzai *et al*. (2001) showed that the purified TRAS1Bm EN specifically nicks the telomeric repeat sequence between the T and A of (TT↑AGG)n. The locations 7 bp upstream and 3 bp downstream from the T-A junction (5′-TTAGGTT↑AGG-3′) have also been shown to be important for the TRAS1Bm EN recognition and the GTTAG sequence is essential for the cleavage reaction on the bottom strand^22^ (Fig. 5a). Here, using an *ex vivo* assay, we found that SART1Bm requires at least three (TTAGG) repeats [upstream 12 bp (3′-AATCCAATCCAA↑-5′)] for retrotranspostion (Figs. 1b and 5b). Furthermore, mutation analysis of the target site revealed that the nucleotide A in the (TTAGG) repeats is essential for retrotransposition (Figs. 3a and 5b). Because of the similarity in structure between TRAS1Bm and SART1Bm, we here assumed that three telomeric repeats and nucleotide A in the bottom strand are also required for SART1Bm endonuclease cleavage. These results also indicate that SART1Bm does not retrotranspose at the end of telomeres like Het or TART elements in *Drosophila*^12^, but retrotransposes into the telomere repeats.

Considering previous findings and the results obtained in this work, we concluded that telomere-specific non-LTR retrotransposons (SART1Bm and TRAS1Bm) recognize two to three short (TTAGG) repeats, suggesting the similarity in their target-site recognition mechanisms (Fig. 5a,b). In addition, the target sequence specificity of both elements was not influenced by flanking sequences of the unrelated region, illustrating that the EN domains of SART1Bm and TRAS1Bm are the primary determinants of the target-site selection. This also suggests that SART1Bm or TRAS1Bm may retrotranspose into various genomic sites containing a short (TTAGG) repeat. However, it is suggested that most of the two elements are site-specifically inserted into the (TTAGG) repeats in the telomere region but not into other regions in the genome. One possible explanation for this result is that SART1Bm and TRAS1Bm proteins might interact with telomere-specific chromatin components or telomere-binding proteins to lead RNP to access specifically to the telomere target site; the EN domain then determines the precise sequence for integration by recognizing about 10 bp of the sequences in both elements.

We also found that SART1Bm retrotransposes into telomeric repeats of other species, not only (TCAGG)_23_ of *T. castaneum*, which was reported previously, but also (TTAGGG)_34_ of *H. sapiens*, (TTAGGC)_23_ of *C. elegans*, and (TTTAGGG)_21_ of *A. thaliana*. However, sequencing of 16 clones for each construct revealed that only one or two clones had undergone retrotransposition compared with 15/16 clones of the host telomere of (TTAGG)_21_, suggesting that SART1Bm preferentially inserts into telomeric repeats of its host, *B. mori*. A previous study also showed that protein-engineered EN of TRAS1Bm combined with telomere-binding proteins, TRF1, cleaves the human telomeric repeat (TTAGGG)_n_ in a sequence-specific manner^27^. Since (TTAGGG)_n_-type telomeric repeats are generally conserved among vertebrates, indicating that SART1Bm may be applicable as a gene delivery tool.

Next, we developed a novel method of *trans-in vivo* retrotransposition assay. This assay showed that SART1Bm ORF proteins mobilize the EGFP/3′ UTR-(A)_n_ donor plasmid *in trans*. As mentioned above, the SART1Bm transcription continues through the poly(A) tract until reaching the vector 3′ regions (Fig. 4a). In human non-LTR retrotransposon L1 elements, the read-through downstream region of its 3′ UTR can be retrotransposed into the genome by recognizing the downstream poly(A) tail. This phenomenon called 3′ transduction was first demonstrated in a cell cultured retrotransposon assay, and subsequent human and mouse genome sequence analyses revealed that the 3′ transduction is a common feature in the genome^28,29^. Furthermore, R7Ag with a shorter poly(A) tail resulted in the 3′ transduction^24^, which was also observed in R1Bm^30^. Although further studies such as using internal PCR are needed to rule out the potential 3’ transduction events, in this assay, we detected no SART1Bm 3′ UTR transduction. However, we did observe the internal reverse transcription from a position -394 internal to the 3′ UTR in donor constructs, even in the poly(A) deleted construct of A-0 (Fig. 4). This indicates that the RT domain of SART1Bm may have higher stringency on the 3′ UTR than does R7Ag.

A previous study showed that in SART1Bm, short telomeric-repeat-like GGUU sequences in the 3′ UTR of mRNA might have annealed to the bottom-strand target-site (TTAGG)_n_ repeats, allowing reverse transcription to initiate from the internal site of the 3′ UTR^26^. In this study, we observed that most SART1Bm 3′ UTR internal initiation started the telomeric-repeat-like AGG sequence at the position of the -394 bp (AGG) in the 3′ UTR of mRNA, which might also anneal to the bottom strand TCC target site (Supplementary Tables 6 and 8).

**Figure 6:**
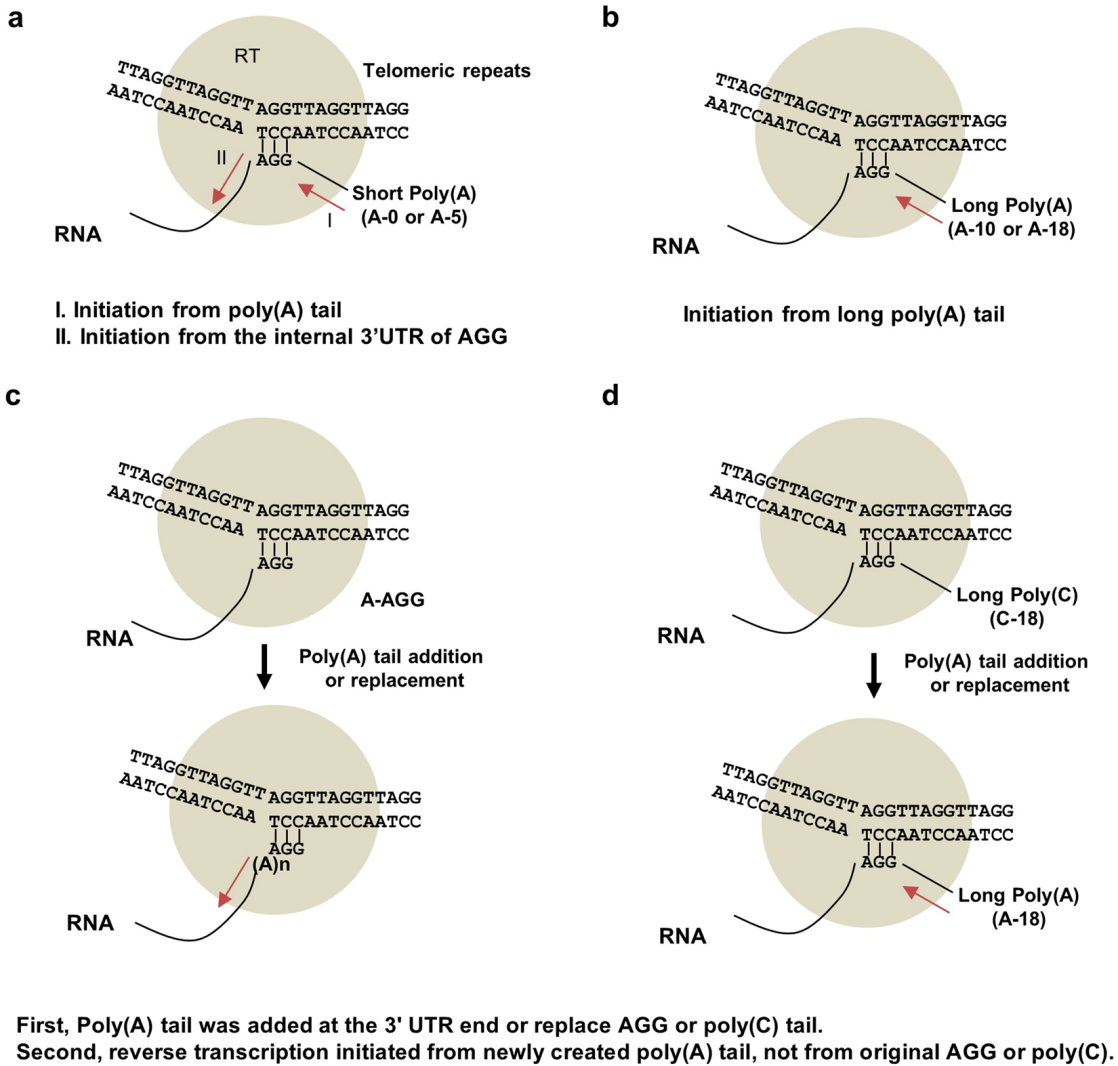
Hypothetical models for the role of poly(A) tail in the process of initiation of reverse transcription in SART1Bm. (**a**) The internal AGG of the SART1Bm 3′ UTR anneals to the bottom strand TCC of the telomeric DNA. The putative interaction is indicated by short black lines. The RT domain is shown as gray circles. During the process of retrotransposition, the RT domain recognizes the 3′ UTR more effectively than a short poly(A) tail; therefore, reverse transcription sometimes starts at the internal region of the 3′ UTR. The cDNA synthesis is shown by a red arrow. (**b**) In longer poly(A) tail constructs (A-10 and A-18), both the 3′ UTR and the longer poly(A) might be recognized more effectively, leading to the accurate insertion of SART1Bm. (**c**) In the AGG construct, the 3′ UTR end of the AGG sequence may anneal to the target site to initiate reverse transcription. (**d**) When the C-18 construct is used, SART1Bm is reverse transcribed from the variable lengths of poly(A) tail but not from the poly(C) tail, indicating that the poly(A) tail may be added to the 3′ UTR end by cellular poly(A) polymerase before reverse transcription.

Based on these observations, we propose a model to explain the role of the poly(A) tail in SART1Bm retrotransposition (Fig. 6). During retrotransposition, RT or some other domain recognizes the 3′ UTR more effectively than a short poly(A) tail in the shorter poly(A) donor constructs; therefore, reverse transcription sometimes starts at the internal region of the 3′ UTR, which may anneal to the target site (Fig. 6a). However, in longer poly(A) tail constructs (A-10 and A-18), both the 3′ UTR and the longer poly(A) might be recognized more effectively, leading to the accurate insertion of SART1Bm (Fig. 6b). The addition of the AGG sequence to the A-0 construct increased the rate of accurate reverse transcription from 36% to 76% (Supplementary Tables 6 and 8).This indicates that the AGG sequence may anneal to the target site to initiate reverse transcription, as in R1Bm^30^, which requires a downstream target site for accurate reverse transcription. When we replaced the poly(A) tail with a poly(C) tail, SART1Bm was reverse transcribed from the variable-length poly(A) tail but not from the poly(C) tail (Fig. 6d). This indicates that the length of the poly(A) tail is more important than the specific sequence. Similar studies reported that, for human L1, a poly(A) tail is critical for L1 retrotransposition *in cis*^31^^,^ and for human Alu elements, longer a poly(A) tail encoded by Alu is required for the efficient retrotransposition *in trans*^32^”.

Another unexpected finding is that the poly(A) tract was located at the boundary between the transposed SART1Bm 3′ UTR and telomeric repeats when using A-0, AGG, and C-18 constructs, which lack the original poly(A) tail downstream of the 3′ UTR (Supplementary Tables 6 and 8). The reason why these poly(A) tails added to the 3′ end of SART1Bm is unclear. This phenomenon was also observed in previous studies of SART1Bm^18^, R2Bm^33^, and L1^31^. One hypothesis suggests that the poly(A) tail is extended at the 3′ end post-transcriptionally by cellular poly(A) polymerase or through a cryptic polyadenylation signal^31^. Alternatively, it is suggested that additional poly(A) may be added by the RT domain before reverse transcription is initiated^33^ (Fig. 6c,d). However, further study is needed to clarify how the poly(A) is added to the end of the 3′ UTR.

## Materials and Methods

### Sf9 cell culture

Sf9 cells (Invitrogen) derived from the pupal ovarian tissue of the fall army worm, *Spodoptera frugiperda*, were maintained at 27 °C in Grace’s Medium, supplemented (Gibco) added to a final concentration of 10% fetal bovine serum (Gibco), 40 units/mL penicillin, and 40 μg/mL streptomycin (Gibco). Cells were under adherent cultures, and passaging was conducted every 72 h, at confluent condition at a 1:5 dilution to maintain log-phase growth.

### Plasmid construction

For plasmid construction, polymerase chain reaction (PCR) was conducted with iProof^TM^ DNA polymerase (Bio-Rad, Hercules, CA, USA). The primers used for plasmid construction are shown in Supplementary Table 1.

### Target plasmid construction for SART1Bm

Target plasmids of (TTAGG)_1_, (TTAGG)_2_, (TTAGG)_3_, (TTAGG)_5_, (TTAGG)_8_, and (TTAGG)_13_ were constructed by annealing oligodeoxynucleotide pairs shown in Supplementary Table 1 by heating at 95 °C for 5 min and then cooled at room temperature. The resulting double-stranded oligos were subcloned to pBluescript II SK (+) by *Eco*RI. The construction of (TTAGG)_21_, (TTAGGG)_34_, (TTAGGC)_23_, and (TTTAGGG)_21_ and point mutation of (TTAGG)_n_-pBSK plasmids [(CTAGG)_42_, (TCAGG)_23_, (TTCGG)_35_, (TTACG)_30_, and (TTAGC)_32_] were carried out using the same method as for TTAGG_25_-pBSK construction^19^. Specifically, to obtain corresponding telomeric repeats, PCR amplification was carried out using a primer set shown in Supplementary Table 1 without a template^34^. The PCR products were cloned by pGEM-T Easy Vector System (Promega, Madison, WI, USA), and the resulting plasmid including target telomeric repeats was isolated. The telomeric repeat sequence was subcloned into pBluescript II SK-(+) by *Eco*RI.

### Enhanced green fluorescence protein (EGFP)/3′ UTR series plasmid

Initially, the pIZT/V5-His-dEGFP plasmid was constructed using a pIZT/V5-His Vector (Invitrogen, Carlsbad, CA, USA) from which EGFP had been deleted. Next, the 3xFLAG-pIZT/V5-His-dEGFP construct was constructed as follows: the 3xFLAG-tag region, which includes KOZAK sequences (GCCACC) and a 3xFLAG tag (DYKDHDGDYKDHDIDYKDDDDK), was constructed by incubating phosphorylated oligodeoxynucleotides with KpnI-KOZAK-3xFLAG-B-S and KpnI-KOZAK-3xFLAG-B-AS primers and T4 Polynucleotide Kinase (Toyobo, Osaka, Japan) at 37 °C for 1 h, followed by 5 min incubation at 95 °C and cooling to room temperature. The final resulting product was subcloned between the *Kpn*I and *Bam*HI sites of pIZT/V5-His-dEGFP. 3xFLAG-EGFP-pIZT/V5-His-dEGFP was constructed as follows: a portion of EGFP was amplified by PCR from the pHSP70 -EGFP-SART1-ORF1-1-447^35^ plasmid with primers BamHI-EGFP-S96 and EcoRI-EGFP-A813. The PCR product was then subcloned between the *Bam*HI and *Eco*RI sites of 3xFLAG-pIZT/V5-His-dEGFP. To construct the variable length of the poly(A) tail SART1Bm plasmid, a portion of SART1Bm/3′ UTR with a variable poly(A) tail was amplified by PCR from the EGFP1/S1-3′ UTR-pVL1393 plasmid^26^ with the primer SART1-S6221-EcoRI-Takahashi and corresponding reverse primer, as shown in Supplementary Table 1. The resulting PCR product was subcloned between the *Eco*RI and *Xba*I sites of 3xFLAG-EGFP-pIZT/V5-His-dEGFP.

### Recombinant AcNPV generation

Recombinant AcNPV generation was performed according to the instructions provided with the Bac-to-Bac Baculovirus Expression System (Invitrogen). Briefly, the above mentioned recombinant pFastBac HTC constructs, which contained a gene of interest driven by the polyhedron promoter, were transformed into DH10Bac *Escherichia. coli* for bacmid transposition. Next, the recombinant bacmid DNA was isolated, and PCR was used to confirm the success of transposition. The isolated recombinant bacmid was then transfected into Sf9 cells using Cellfectin® Reagent (Invitrogen) according to the instruction manual. Four days later, the medium containing the virus was collected and centrifuged at 500 g for 5 min to remove cells and large debris. The clarified supernatant was transferred to fresh tubes and designated P1 viral stock for use in plaque assays and viral amplification. SART1Bm baculovirus is the same as SART1WT-pAcGHLTB as reported in the paper by Takahashi and Fujiwara (2002).

### *Ex vivo* retrotransposition assay

The *ex vivo* retrotransposition assay was performed as follows. Approximately 3 × 10^5^ Sf9 cells in a 12-well plate were transfected with 800 ng of the target plasmid with the TransFast™ Transfection Reagent (Promega). Subsequently, these cells were infected with SART1Bm at MOI1. Plasmid DNA was extracted 72 h after infection. The PCR assay was conducted for SART1Bm with *Ex-Taq* (TaKaRa, Shiga, Japan). PCR was denatured at 94 °C for 1 min, followed by 35 cycles of 94 °C for 20 s, 60 °C for 30 s, and 72 °C for 50 s. The primers used for the nested PCR assay are shown in Supplementary Table 1. The PCR products were directly cloned into the pGEM-T Easy vector (Promega). The cloned products were sequenced with a BigDye Terminator cycle sequencing kit (Applied Biosystems, Foster City, CA, USA) on ABI 3130xl and 3500/3500xl Genetic Analyzers (Applied Biosystems). Sequence analysis was performed using the Vector NTI Advance 10 system (Invitrogen).

### *Trans-in vivo* retrotransposition assay

To develop the *trans-in vivo* retrotransposition assay, modification of the *in vivo* retrotransposition assay was performed as follows. Approximately 1 × 10^6^ Sf9 cells in a 6-well plate were transfected with 800 ng of each SART1Bm plasmid with variable lengths of poly(A) for 3 h. Then, these cells were infected with SART1Bm AcNPV at MOI1. The genomic DNA was extracted 72 h post-infection with Gentra Puregene® Kits (QIAGEN, Valencia, CA, USA). PCR assays were conducted using *Ex-Taq* with 1 μg of Sf9 DNA and the primers pEGFP1-S688 and CCTAA6. The reaction mixture was denatured at 96 °C for 2 min, followed by 35 cycles of 96 °C for 30 s, 62 °C for 30 s, and 72 °C for 1 min. One microliter of each mixture was subjected to 2% agarose electrophoresis in Tris-acetate-EDTA buffer and visualized by ethidium bromide staining. The PCR products were directly cloned into the pGEM-T Easy vector (Promega). The cloned products were sequenced and analyzed.

## Supplementary information

Supplementary Material Tables.

## Data Availability

All data generated or analyzed during this study are included in this published article and its Supplementary Information Files.
