# Supplementary material for: Essential factors involved in the precise targeting and insertion of telomere-specific non-LTR retrotransposon, SART1Bm
Source: Sci Rep. 2020 Jun 2;10:8963. doi: 10.1038/s41598-020-65925-x (PMC7265360; doi:10.1038/s41598-020-65925-x)
Supplement: Supplementary file 1 — Supplementary Material Tables. [file 41598_2020_65925_MOESM1_ESM.pdf]

Supplementary Material

Essential factors involved in the precise targeting and insertion of telomere-specific non-LTR retrotransposon, SART 1Bm

Authors:

Narisu Nichuguti* and Haruhiko Fujiwara*[1]

Affiliations:

*Department of Integrated Biosciences, Graduate School of Frontier Sciences, The University of Tokyo, Kashiwa, Chiba, 277-8562, Japan.

[1]**Corresponding author**: Department of Integrated Biosciences, Graduate School of Frontier Sciences, The University of Tokyo, Bioscience Bldg., Kashiwanoha 5-1-5, Kashiwa, Chiba, 277-8562, Japan.

Tel: +81-4-7136-3659; Fax: +81-4-7136-3660;

Email: haruh@edu.k.u-tokyo.ac.jp

Supplementary Figure S1 Full-length gels regarding data shown in Fig. 1b

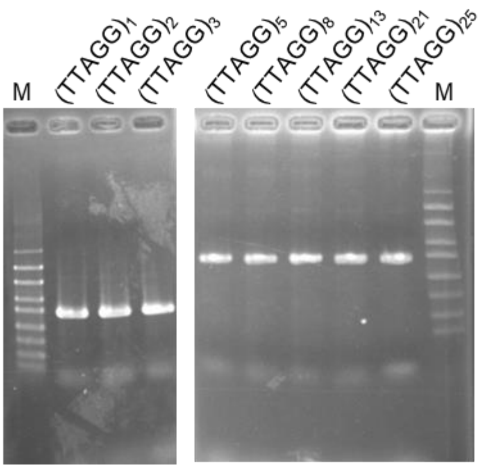

Supplementary Table 1. Primers used in this study

Name	Sequence (5′ to 3′)
**SART1Bm target plasmid construction**	
E-TTAGG1-S-E-s	AATTCGATTTTAGGAATCACTAGTG
E-TTAGG1-S-E-a	AATTCACTAGTGATTCCTAAAATCG
E-TTAGG2-S-E-s	AATTCGATTTTAGGTTAGGAATCACTAGTG
E-TTAGG2-S-E-a	AATTCACTAGTGATTCCTAACCTAAAATCG
E-TTAGG3-S-E-s	AATTCGATTTTAGGTTAGGTTAGGAATCACTAGTG
E-TTAGG3-S-E-a	AATTCACTAGTGATTCCTAACCTAACCTAAAATCG
E-TTAGG5-S-E-s	AATTCGATTTTAGGTTAGGTTAGGTTAGGTTAGGAATCACTAGTG
E-TTAGG5-S-E-a	AATTCACTAGTGATTCCTAACCTAACCTAACCTAACCTAAAATCG
E-TTAGG8-S-E-s	AATTCGATTTTAGGTTAGGTTAGGTTAGGTTAGGTTAGGTTAGGTTAGG AATCACTAGTG
E-TTAGG8-S-E-a	AATTCACTAGTGATTCCTAACCTAACCTAACCTAACCTAACCTAACCTAA CCTAAAATCG
E-TTAGG13-S-E-s	AATTCGATTTTAGGTTAGGTTAGGTTAGGTTAGGTTAGGTTAGGTTAGG TTAGGTTAGGTTAGGTTAGGTTAGGAATCACTAGTG
E-TTAGG13-S-E-a	AATTCACTAGTGATTCCTAACCTAACCTAACCTAACCTAACCTAACCTAA CCTAACCTAACCTAACCTAACCTAACCTAAAATCG
(TTAGG)6	TTAGGTTAGGTTAGGTTAGGTTAGGTTAGG
(CCTAA)6	CCTAACCTAACCTAACCTAACCTAACCTAA
(CTAGG)5	CTAGGCTAGGCTAGGCTAGGCTAGG
(CCTAG)5	CCTAGCCTAGCCTAGCCTAGCCTAG
(TCAGG)5	TCAGGTCAGGTCAGGTCAGGTCAGG
(CCTGA)5	CCTGACCTGACCTGACCTGACCTGA
(TTCGG)5	TTCGGTTCGGTTCGGTTCGGTTCGG
(CCGAA)5	CCGAACCGAACCGAACCGAACCGAA
(TTACG)5	TTACGTTACGTTACGTTACGTTACG
(CGTAA)5	CGTAACGTAACGTAACGTAACGTAA
(TTAGC)5	TTAGCTTAGCTTAGCTTAGCTTAGC
(GCTAA)5	GCTAAGCTAAGCTAAGCTAAGCTAA
(TTAGGG)5	TTAGGGTTAGGGTTAGGGTTAGGGTTAGGG
(CCCTAA)5	CCCTAACCCTAACCCTAACCCTAACCCTAA
(TTAGGC)4	TTAGGCTTAGGCTTAGGCTTAGGC
(GCCTAA)4	GCCTAAGCCTAAGCCTAAGCCTAA

(TTTAGGG)3	TTTAGGGTTTAGGGTTTAGGG
(CCCTAAA)3	CCCTAAACCCTAAACCCTAAA
**EGFP/3′UTR series plasmid**	
BamHI-EGFP-S96	AAGGATCCATGGTGAGCAAGGGCGAGG
EcoRI-EGFP-A813	AAGAATTCTTACTTGTACAGCTCGTCCATGC
SART1-S6221-EcoRI-Takahashi	TTTTTGAATTCGGACCGTCGGGCGTC
TTT-XbaI-PolyA(0)+SART1 3UTR	TTTtctagaGGTATCGATGGGGAATCCC
TTT-XbaI-PolyA(5)+SART1 3UTR	TTTtctagaTTTTTGGTATCGATGGGGAATCCC
TTT-XbaI-PolyA(10)+SART1 3UTR	TTTtctagaTTTTTTTTTTGGTATCGATGGGGAATCCC
TTT-XbaI-PolyA(18)+SART1 3UTR	TTTtctagaTTTTTTTTTTTTTTTTTTGGTATCGATG
TTT-XbaI-PolyC(18)+SART1 3UTR	TTTtctagaGGGGGGGGGGGGGGGGGGGGTATCGATGGGGAATCCC
TTT-XbaI-PolyA(0)AGG+SART1 3UTR	TTTtctagaCCTGGTATCGATGGGGAATCCC
***Ex vivo* retrotransposition assay**	
S16131	AGAAAGAGAGTGCGACCCAAACTCAGTT
A878T	GGCTGCAGGAATTCACTAGTGATT
Amp-F1	GAACGTTTTCCAATGATGAGCACTT
Amp-R1	CCAATGCTTAATCAGTGAGGCACC
***Trans-in vivo* retrotransposition assay**	
pEGFP1-S688	GACAACCACTACCTGAGCACC
CCTAA6	CCTAACCTAACCTAACCTAA CCTAACCTAA

Supplementary Table 2. Artifactual baculovirus sequences detected in *ex vivo* assay.

Type	Query Length(bp)	Sbjct (bp)	Identities	Synthetic baculovirus AcMNPV-WIV-Syn1, complete sequence (ID: KY792989.1)		
				**Forward primer: S16131**<br>**AGAAAGAGAGTGCGACCCAAACTCAGTT**	**Baculovrisu p10 and p74 genes' junction region**	**Reverse Primer: A878T**<br>**GGCTGCA-GGAATTCACTAGTGATT**
1	398 (8-398)	36785 to 37175	391/391(100%)	AGAAAGAGAGTGCGACCCAAACTCACTT	GCACTCAGGCGAAAGAGACGGATTTTTAATCAAATAATCATGGGTTTATTTGCAACAAACACAATTGTGGCGTTTACAGCA<br>TTTGTTATACACACAGAACTCATATTTTTATATTTTTCGTAATCTTCCTAATGATCACATTTTATTACATAATCAAAGAA<br>TCGTACGAATATTATAAAACAATTGATTTGTTATTTTAAAAACGATTCATTTACTTGGAACTGCGTTTACCACGACGAGCG<br>TCTGAATCGAGTTCAAAAGCTTGGCTTTTCAGCTTAGGCTTTAGTGAGTCTGGAAGATCCGGAACAATGTCGCCGGTCAAT<br>ATGGATTGAATTTCTGAGATCTTAGTGTTAAGATCGGTCAATTGAGCGGGCAAACCGTCCAAAAGCTGGAATGATTCTTCC<br>AGCCCGTTTAACTGAGTTTGGGTCGCACTCTCTTTCT	AATCACTAGTGAATTCGCGGCCGCC
2	425 (39-425)	36785 to 37171	387/387(100%)	AGAAAGAGAGTGCGACCCAAACTCAGTT	GCACTCAGGCGAAAGAGACGACACCTCCCAACCGCAGACGCGGCGCGCGACGTCAATCAAATAATCATGGGTTTATTTGCA<br>ACAAACACAATTGTGGCGTTTACAGCATTTGTTATACACACAGAACTCATATTTTTATATTTTTCGTAATCTTCCTAATG<br>ATCACATTTTATTACATAATCAAAGAATCGTACGAATATTATAAAACAATTGATTTGTTATTTTAAAAACGATTCATTTAC<br>TTGGAACTGCGTTTACCACGACGAGCGTCTGAATCGAGTTCAAAAGCTTGGCTTTTCAGCTTAGGCTTTAGTGAGTCTGGA<br>AGATCCGGAACAATGTCGCCGGTCAATATGGATTGAATTTCTGAGATCTTAGTGTTAAGATCGGTCAATTGAGCGGGCAAA<br>CCGTCCAAAAGCTGGAATGATTCTTCCAGCCCGTTTAACTGAGTTTGGGTCGCACTCTCTTTCT	AATCACTAGTGAATTCGCGGCCGCC
3	378 (1-375)	36800 to 37174	374/375(99%)	AGAAAGAGAGTGCGACCCAAACTCAGTT	GCACTCAGGCGAAAGATCATTCCAGCTTTTGGACGGTTTGCCCGCTCAATTGACCGATCTTAACACTAAGATCTCAGAAAT<br>TCAATCCATATTGACCGGCGACATTGTTCCGGATCTTCCAGACTCACTAAAGCCTAAGCTGAAAAGCCAAGCTTTTGAACT<br>CGATTCAGACGCTCGTCGTGGTAAACGCAGTTCCAAGTAAATGAATCGTTTTTAAAATAACAAATCAATTGTTTTATAATA<br>TTCGTATGATTCTTTGATTATGTAATAAAATGTGATCATTAGGAAGATTACGAAAAATATAAAAAATATGAGTTCTGTGTG<br>TATAACAAATGCTGTAAACGCCACAATTGTGTTTGTTGCAAATAAACCCATGATTATTTGATTAAACGCAACTGAGTTTGG<br>GTCGCACTCTCTTTCT	AATCACTAGTGAATTCGCGGCCGCC
4	439 (1-364)	36774 to 37163	363/364(99%)	AGAAAGAGAGTGCGACCCAAACTCAGTT	GCACTCAGGCGAAAGATCATTCCAGCTTTTGGACGGTTTGCCCGCTCAATTGACCGATCTTAACACTAAGATCTCAGAAAT<br>TCAATCCATATTGACCGGCGACATTGTTCCGGATCTTCCAGACCCACTAAAGCCTAAGCTGAAAAGCCAAGCTTTTGAACT<br>CGATTCAGACGCTCGTCGTGGTAAACGCAGTTCCAAGTAAATGAATCGTTTTTAAAATAACAAATCAATTGTTTTATAATA<br>TTCGTACGATTCTTTGATTATGTAATAAAATGTGATCATTAGGAAGATTACGAAAAATATAAAAAATATGAGTTCTGTGTG<br>TATAACAAATGCTGTAAACGCCACAATTGTGTTTGTTGCAAATAAACCCATGATTGGTCCAGCTGAGCGGGGGCCCTTCGC<br>CCGCGCCGCCCACCCCGTCTCTTACGCCTGAGTGCAACTGAGTTTGGGTAACTGAGTTTGGGTCGCACTCTCTTTCT	AATCACTAGTGAATTCGCGGCCGCC

				**Reverse Primer: A878T**<br>**GGCTGCA-GGAATTCACTAGTGATT**		**Forward primer: S16131**<br>**AGAAAGAGAGTGCGACCCAAACTCAGT**
5	441 (59-441)	36785 to 37167	383/383(100%)	GGCGGCCGCGAATTCACTAGTGATT	AGAAAGAGAGTGCGACCCAAACTCAGTTGCACTCAGGCGAAAGAGACGGGGTGGGCGGCGCGGGCGAAGGGCCCCCGCTCA<br>GCTCAAAATAATCATGGGTTTATTTGCAACAAACACAATTGTGGCGTTTACAGCATTTGTTATACACACAGAACTCATATT<br>TTTTATATTTTTCGTAATCTTCCTAATGATCACATTTTATTACATAATCAAAGAATCGTACGAATATTATAAAACAATTGA<br>TTTGTTATTTTAAAAACGATTCATTTACTTGGAACTGCGTTTACCACGACGAGCGTCTGAATCGAGTTCAAAAGCTTGGCT<br>TTTCAGCTTAGGCTTTAGTGAGTCTGGAAGATCCGGAACAATGTCGCCGGTCAATATGGATTGAATTTCTGAGATCTTAGT<br>GTTAAGATCGGTCAATTGAGCGGGCAAACCGTCCAAAAGCTGGAATGATTCTTCCAGCCCGTTT	AACTGAGTTTGGGTCGCACTCTCTTTCT
6	393 (4-393)	36785 to 37174	389/390(99%)	GGCGGCCGCGAATTCACTAGTGATT	AGAAAGAGAGTGCGACCCAAACTCAGTTGCGTTTAATCAAATAATCATGGGTTTATTTGCAACAAACACAATTGTGGCGTT<br>TACAGCATTTGTTATACACACAGAACTCATATTTTTATATTTTTCGTAATCTTCCTAATGATCACATTTTATTACATAAT<br>CAAAGAATCATACGAATATTATAAAACAATTGATTTGTTATTTTAAAAACGATTCATTTACTTGGAACTGCGTTTACCACG<br>ACGAGCGTCTGAATCGAGTTCAAAAGCTTGGCTTTTCAGCTTAGGCTTTAGTGAGTCTGGAAGATCCGGAACAATGTCGCC<br>GGTCAATATGGATTGAATTTCTGAGATCTTAGTGTTAAGATCGGTCAATTGAGCGGGCAAACCGTCCAAAAGCTGGAATGA<br>TTCTTCCAGCCCGTTT	AACTGAGTTTGGGTCGCACTCTCTTTCT
7	442 (1-384)	36785 to 37167	381/384(99%)	GGCGGCCGCGAATTCACTAGTGATT	AGAAAGAGAGTGCGACCCAAACTCAGTTAAACGGGCTGGAAGAATCATTCCANNTTTTTGGACGGTTTGCCCGCTCAATTG<br>ACCGATCTTAACACTAAGATCTCAGAAATTCAATCCATATTGACCGGCGACATTGTTCCGGATCTTCCAGACTCACTAAAG<br>CCTAAGCTGAAAAGCCAAGCTTTTGAACTCGATTCAGACGCTCGTCGTGGTAAACGCAGTTCCAAGTAAATGAATCGTTTT<br>TAAAATAACAAATCAATTGTTTTATAATATTCGTACGATTCTTTGATTATGTAATAAAATGTGATCATTAGGAAGATTACG<br>AAAAATATAAAAAATATGAGTTCTGTGTGTATAACAAATGCTGTAAACGCCACAATTGTGTTTGTTGCAAATAAACCCATG<br>ATTATTTTGAGCTGAGCGGGGGCCCTTCGCCCGCGCCGCCCACCCCGTCTCTTTCGCCTGAGTGC	AACTGAGTTTGGGTCGCACTCTCTTTCT
8	456 (1-390)	36785 to 37175	390/391(99%)	GGCGGCCGCGAATTCACTAGTGATT	AGAAAGAGAGTGCGACCCAAACTCAGTTAAACGGGCTGGAAGAATCATTCCAGCTTTTGGACGGTTTGCCCGCTCAATTGA<br>CCGATCTTAACACTAAGATCTCAGAAATTCAATCCATATTGACCGGCGACATTGTTCCGGATCTTCCAGACTCACTAAAGC<br>CTAAGCTGAAAAGCCAAGCTTTTGAACTCGATTCAGACGCTCGTCGTGGTAAACGCAGTTCCAAGTAAATGAATCGTTTTT<br>AAAATAACAAATCAATTGTTTTATAATATTCGTACGATTCTTTGATTATGTAATAAAATGTGATCATTAGGAAGATTACGA<br>AAAATATAAAAAATATGAGTTCTGTGTGTATAACAAATGCTGTAAACGCCACAATTGTGTTTGTTGCAATAAACCCATGAT<br>TATTTGATTAAAAAAAAAAAAAAAAGCTGAGCGGGGGCCCTTCGCCCGCGCCGCCCACCCCGTCTCTTTCGTCTGAGTGC	AACTGAGTTTGGGTCGCACTCTCTTTCT
9	426 (1-392)	36785 to 37175	390/392(99%)	GGCGGCCGCGAATTCACTAGTGATT	AGAAAGAGAGTGCGACCCAAACTCAGTTAAACGGGCTGGAAGAATCATTCCAGTNTTTTGGACGGTTTGCCCGCTCAATTG<br>ACCGATCTTAACACTAAGATCTCAGAAATTCAATCCATATTGACCGGCGACATTGTTCCGGATCTTCCAGACTCACTAAAG<br>CCTAAGCTGAAAAGCCAAGCTTTTGAACTCGATTCAGACGCTCGTCGTGGTAAACGCAGTTCCAAGTAAATGAATCGTTTT<br>TAAAATAACAAATCAATTGTTTTATAATATTCGTACGATTCTTTGATTATGTAATAAAATGTGATCATTAGGAAGATTACG<br>AAAAATATAAAAAATATGAGTTCTGTGTGTATAACAAATGCTGTAAACGCCACAATTGTGTTTGTTGCAAATAAACCCATG<br>ATTATTTGATTAAAAAAAAAAAAAAAAAAAAAAAAAAAAAAAAAAAGTGC	AACTGAGTTTGGGTCGCACTCTCTTTCT
10	391 (1-390)	36785 to 37174	388/390(99%)	GGCGGCCGCGAATTCACTAGTGATT	AAAGAGAGTGCGACCCAAACTCAGTTAAACGGGCTGGAAGAATCATTCCAGCTTTTGGACGGTTTGCCCGCTCAATTGACC<br>GATCTTAACACTAAGATCTCAGAAATTCAATCCATATTGACCGGCGACATTGTTCCGGATCTTCCAGACTCACTAAAGCCT<br>AAGCTGAAAAGCCAAGCTTTTGAACTCGATTCAGACGCTCGTCGTGGTAAACGCAGTTCCAAGTAAATGAATCGTTTTTAA<br>AATAACAAATCAATTGTTTTATAATATTCGTATGATTCTTTGATTATGTAATAAAATGTGATCATTAGGAAGATTACGAAA<br>AATATAAAAAATATGAGTTCTGTGTGTATAACAAATGCTGTAAACGCCTCAATTGTGTTTGTTGCAAATAAACCCATGATT	AACTGAGTTTGGGTCGCACTCTCTTTCT
11	455 (1-392)	36785 to 37175	387/392(99%)	GGCGGCCGCGAATTCACTAGTGATT	AGAAAGAGAGTGCGACCCAAACTCAGTTAAACGGGCTGGAAGAATCATTCCNTNNTTTTGGACGGTTTGCCCGCTCAATTG<br>ACCGATCTTAACACTAAGATCTCAGAAATTCAATCCATATTGACCGGCGACATTGTTCCGGATCTTCCAGACTCACTAAAG<br>CCTAAGCTGAAAAGCCAAGCTTTTGAACTCGATTCAGACGCTCGTCGTGGTAAACGCAGTTCCAAGTAAATGAATCGTTTT<br>TAAAATAACAAATCAATTGTTTTATAATATTCGTACGATTCTTTGATTATGTAATAAAATGTGATCATTAGGAAGATTACG<br>AAAAATATAAAAAATATGAGTTCTGTGTGTATAACAAATGCTGTAAACGCCACAATTGTGTTTGTTGCAAATAAACCCATG<br>ATTGTTTGATTAAAAAAAAAAAAAAAAGCTGAGCGGGGGCCCTTCGCCCGCGCCGCCCACCCCGTCTCTTTCGTCTGAGTGC	AACTGAGTTTGGGTCGCACTCTCTTTCT
12	412 (1-391)	36785 to 37175	390/391(99%)	GGCGGCCGCGAATTCACTAGTGATT	AGAAAGAGAGTGCGACCCAAACTCAGTTAAACGGGCTGGAAGAATCATTCCAGCTTTTGGACGGTTTGCCCGCTCAATTGA<br>CCGATCTTAACACTAAGATCCCAGAAATTCAATCCATATTGACCGGCGACATTGTTCCGGATCTTCCAGACTCACTAAAGC<br>CTAAGCTGAAAAGCCAAGCTTTTGAACTCGATTCAGACGCTCGTCGTGGTAAACGCAGTTCCAAGTAAATGAATCGTTTTT<br>AAAATAACAAATCAATTGTTTTATAATATTCGTACGATTCTTTGATTATGTAATAAAATGTGATCATTAGGAAGATTACGA<br>AAAATATAAAAAATATGAGTTCTGTGTGTATAACAAATGCTGTAAACGCCACAATTGTGTTTGTTGCAAATAAACCCATGA<br>TTATTTGATTAAAAACCCGTCTCTTTCGCCTGAGTGC	AACTGAGTTTGGGTCGCACTCTCTTTCT

Supplementary Table 3. Nucleotide sequence of the 3′ junction of *ex vivo* retrotransposed SART1Bm and point mutated (TTAGG) tracts

Lane	Target plasmid	SART1Bm 3′ UTR/(A)$_n$	Telomeric repeats	Vector sequence	Clones
2	(**C**TAGG)$_{42}$	---CATCGATACC(A)$_{30}$	AGG(CTAGG)$_{22}$	AATCACTAGT---	1
		---CATCGATACC(A)$_{54}$	AGG(CTAGG)$_{9}$	AATCACTAGT---	1
		---CATCGATACC(A)$_{29}$	AGG(CTAGG)$_{29}$	AATCACTAGT---	1
		---CATCGATACC(A)$_{37}$	AGG(CTAGG)$_{32}$	AATCACTAGT---	1
		---CATCGATACC(A)$_{29}$	AGG(CTAGG)$_{32}$	AATCACTAGT---	1
3	(T**C**AGG)$_{23}$	---CATCGATACC(A)$_{36}$	AGG(TCAGG)$_{19}$	AATCACTAGT---	1
		---CATCGATACC(A)$_{10}$	AGG(TCAGG)$_{11}$	AATCACTAGT---	1
		---CATCGATACC(A)$_{23}$	AGG(TCAGG)$_{3}$	AATCACTAGT---	1
		---CATCGATACC(A)$_{25}$	AGG(TCAGG)$_{9}$	AATCACTAGT---	1
4	(TT**C**GG)$_{35}$				0
5	(TTA**C**G)$_{30}$	---CATCGATACC(A)$_{47}$	ACG(TTACG)$_{9}$	AATCACTAGT---	1
		---CATCGATACC(A)$_{18}$	ACG(TTACG)$_{19}$	AATCACTAGT---	1
		---CATCGATACC(A)$_{15}$	ACG(TTACG)$_{14}$	AATCACTAGT---	1
6	(TTAG**C**)$_{32}$	---CATCGATACC(A)$_{20}$	AGC(TTAGC)$_{12}$	AATCACTAGT---	1
		---CATCGATACC(A)$_{36}$	AGC(TTAGC)$_{12}$	AATCACTAGT---	1
		---CATCGATACC(A)$_{31}$	AGC(TTAGC)$_{3}$	AATCACTAGT---	1

Supplementary Table 4. Nucleotide sequence of the 3′ junction of *ex vivo* retrotransposed SART1Bm and telomeric repeats of other species

Lane	Target plasmid	SART1Bm 3′ UTR/(A)$_n$	Telomeric repeats	Vector sequence	Clones
2	(TTAGGG)$_{34}$	---CATCGATACC(A)$_{29}$	AGGG(TTAGGG)$_{31}$	AATCACTAGT---	1
		---CATCGATACC(A)$_{18}$	AGGG(TTAGGG)$_{7}$	AATCACTAGT---	1
3	(TTAGGC)$_{23}$	---CATCGATACC(A)$_{14}$	AGGC(TTAGGC)$_{12}$	AATCACTAGT---	1
4	(TTTAGGG)$_{21}$	---CATCGATACC(A)$_{29}$	AGGG(TTTAGGG)$_{3}$	AATCACTAGT---	1
		---CATCGATACC(A)$_{17}$	AGGG(TTTAGGG)$_{2}$	AATCACTAGT---	1
		---CATCGATACC(A)$_{27}$	AGGG(TTTAGGG)$_{11}$	AATCACTAGT---	1

Supplementary Table 5. Nucleotide sequence of 3′ junction clones obtained from the EGFP/ SART1 3' UTR/(A)$_n$ construct.

Reporter	EGFP/*Eco*RI	SART1Bm 3' UTR/(A)$_n$	Telomeric repeats	6
b	---TACAAGTAAgaattc	GGACCGT---GATACC(A)$_{16}$	AGG(TTAGG)$_7$	1
	---TACAAGTAAgaattc	GGACCGT---GATACC(A)$_{29}$	AGG(TTAGG)$_{32}$	1
	---TACAAGTAAgaattc	GGACCGT---GATACC(A)$_{18}$	AGG(TTAGG)$_{57}$	1
	---TACAAGTAAgaattc	GGACCGT---GATACC(A)$_7$	AGG(TTAGG)$_8$	1
	---TACAAGTAAgaattc	GGACCGT---GATACC(A)$_{21}$	AGG(TTAGG)$_{27}$	1
	---TACAAGTAAgaattc	GGACCGT---GATACC(A)$_{14}$	AGG(TTAGG)$_{12}$	1

Supplementary Table 6. Nucleotide sequence of 3′ junction clones obtained from the A-0 and A-5 donor constructs

Reporter	EGFP/*Eco*RI	SART1Bm 3′ UTR	Telomeric repeats	Clone
A-0	---TACAAGTAAgaattc	GGACCGT---TGAGG^{-394*}	AGG (TTAGG)$_5$	1
	---TACAAGTAAgaattc	GGACCGT---TGAGG^{-394*}	AGG (TTAGG)$_6$	1
	---TACAAGTAAgaattc	GGACCGT---TGAGG^{-394*}	AGG (TTAGG)$_{12}$	1
	---TACAAGTAAgaattc	GGACCGT---TGAGG^{-394*}	AGG (TTAGG)$_{38}$	1
	---TACAAGTAAgaattc	GGACCGT---TGAGG^{-394*}	AGG (TTAGG)$_{41}$	1
	---TACAAGTAAgaattc	GGACCGT---TT^{-398}	AGG(TTAGG)$_8$	1
	---TACAAGTAAgaattc	GGACCGT---GATACC(A)$_{16}$	AGG(TTAGG)$_8$	1
	---TACAAGTAAgaattc	GGACCGT---GATACC(A)$_{18}$	AGG(TTAGG)$_9$	1
	---TACAAGTAAgaattc	GGACCGT---GATACC(A)$_{17}$	AGG(TTAGG)$_{10}$	1
	---TACAAGTAAgaattc	GGACCGT---GATACC(A)$_{13}$	AGG(TTAGG)$_{10}$	1
	---TACAAGTAAgaattc	GGACCGT---GATACC(A)$_{27}$	AGG(TTAGG)$_{15}$	1
A-5	---TACAAGTAAgaattc	GGACCGT---TGAGG^{-394*}	AGG (TTAGG)$_6$	1
	---TACAAGTAAgaattc	GGACCGT---TGAGG^{-394*}	AGG (TTAGG)$_9$	1
	---TACAAGTAAgaattc	GGACCGT---GATACC(A)$_{17}$	AGG(TTAGG)$_1$	1
	---TACAAGTAAgaattc	GGACCGT---GATACC(A)$_{24}$	AGG(TTAGG)$_6$	1
	---TACAAGTAAgaattc	GGACCGT---GATACC(A)$_{20}$	AGG(TTAGG)$_6$	1
	---TACAAGTAAgaattc	GGACCGT---GATACC(A)$_{15}$	AGG(TTAGG)$_8$	1
	---TACAAGTAAgaattc	GGACCGT---GATACC(A)$_{20}$	AGG(TTAGG)$_9$	1
	---TACAAGTAAgaattc	GGACCGT---GATACC(A)$_{24}$	AGG(TTAGG)$_{11}$	1
	---TACAAGTAAgaattc	GGACCGT---GATACC(A)$_{23}$	AGG(TTAGG)$_{12}$	1
	---TACAAGTAAgaattc	GGACCGT---GATACC(A)$_{21}$	AGG(TTAGG)$_{16}$	1

*Nucleotide positions are indicated with the junction of cleavage target site AGG and 3'UTR end, defined as 0. The telomeric repeat-like sequences AGG in the end of inserted copies are underlined and indicated at upstream -394.

Supplementary Table 7. Nucleotide sequence of 3′ junction clones obtained from the A-10 and A-18 donor constructs

Reporter	EGFP/*Eco*RI	SART1Bm 3′ UTR	Telomeric repeats	Clone
A-10	---TACAAGTAAgaattc	GGACCGT---GATACC(A)$_{26}$	AGG(TTAGG)$_{1}$	1
	---TACAAGTAAgaattc	GGACCGT---GATACC (A)$_{28}$	AGG(TTAGG)$_{1}$	1
	---TACAAGTAAgaattc	GGACCGT---GATACC (A)$_{20}$	AGG(TTAGG)$_{3}$	1
	---TACAAGTAAgaattc	GGACCGT---GATACC (A)$_{28}$	AGG(TTAGG)$_{5}$	1
	---TACAAGTAAgaattc	GGACCGT---GATACC(A)$_{10}$	AGG(TTAGG)$_{12}$	1
	---TACAAGTAAgaattc	GGACCGT---GATACC (A)$_{29}$	AGG(TTAGG)$_{21}$	1
	---TACAAGTAAgaattc	GGACCGT---GATACC (A)$_{29}$	AGG(TTAGG)$_{25}$	1
	---TACAAGTAAgaattc	GGACCGT---GATACC (A)$_{20}$	AGG(TTAGG)$_{30}$	1
	---TACAAGTAAgaattc	GGACCGT---GATACC (A)$_{11}$	AGG(TTAGG)$_{55}$	1
	---TACAAGTAAgaattc	GGACCGT---GATACC (A)$_{29}$	AGG(TTAGG)$_{65}$	1
A-18	---TACAAGTAAgaattc	GGACCGT---GATACC (A)$_{16}$	AGG(TTAGG)$_{7}$	1
	---TACAAGTAAgaattc	GGACCGT---GATACC (A)$_{7}$	AGG(TTAGG)$_{8}$	1
	---TACAAGTAAgaattc	GGACCGT---GATACC (A)$_{17}$	AGG(TTAGG)$_{8}$	1
	---TACAAGTAAgaattc	GGACCGT---GATACC (A)$_{29}$	AGG(TTAGG)$_{9}$	1
	---TACAAGTAAgaattc	GGACCGT---GATACC (A)$_{14}$	AGG(TTAGG)$_{12}$	1
	---TACAAGTAAgaattc	GGACCGT---GATACC (A)$_{9}$	AGG(TTAGG)$_{22}$	1
	---TACAAGTAAgaattc	GGACCGT---GATACC (A)$_{12}$	AGG(TTAGG)$_{22}$	1
	---TACAAGTAAgaattc	GGACCGT---GATACC (A)$_{21}$	AGG(TTAGG)$_{27}$	1
	---TACAAGTAAgaattc	GGACCGT---GATACC (A)$_{29}$	AGG(TTAGG)$_{32}$	1
	---TACAAGTAAgaattc	GGACCGT---GATACC (A)$_{18}$	AGG(TTAGG)$_{57}$	1

Supplementary Table 8. Nucleotide sequence of 3′ junction clones obtained from the A-AGG and C-18 donor constructs

Reporter	EGFP/*Eco*RI	SART1Bm 3′ UTR	Telomeric repeats	Clone
AGG	---TACAAGTAAgaattc	GGACCGT---TGAGG^{-394*}	AGG $(TTAGG)_6$	1
	---TACAAGTAAgaattc	GGACCGT---TGAGG^{-394*}	AGG $(TTAGG)_9$	1
	---TACAAGTAAgaattc	GGACCGT---GATACC $(A)_{15}$	AGG$(TTAGG)_5$	1
	---TACAAGTAAgaattc	GGACCGT---GATACC $(A)_{17}$	AGG$(TTAGG)_6$	1
	---TACAAGTAAgaattc	GGACCGT---GATACC $(A)_{16}$	AGG$(TTAGG)_3$	1
	---TACAAGTAAgaattc	GGACCGT---GATACC $(A)_{24}$	AGG$(TTAGG)_{12}$	1
	---TACAAGTAAgaattc	GGACCGT---GATACC $(A)_{23}$	AGG$(TTAGG)_{12}$	1
	---TACAAGTAAgaattc	GGACCGT---GATACC $(A)_{16}$	AGG$(TTAGG)_{54}$	1
C-18	---TACAAGTAAgaattc	GGACCGT---ATAGG^{-233*}	AGG $(TTAGG)_8$	1
	---TACAAGTAAgaattc	GGACCGT---GATACC $(A)_{14}$	AGG$(TTAGG)_4$	1
	---TACAAGTAAgaattc	GGACCGT---GATACC $(A)_{62}$	AGG$(TTAGG)_8$	1
	---TACAAGTAAgaattc	GGACCGT---GATACC $(A)_{29}$	AGG$(TTAGG)_8$	1
	---TACAAGTAAgaattc	GGACCGT---GATACC $(A)_{24}$	AGG$(TTAGG)_8$	1
	---TACAAGTAAgaattc	GGACCGT---GATACC $(A)_8$	AGG$(TTAGG)_{16}$	1
	---TACAAGTAAgaattc	GGACCGT---GATACC $(A)_{19}$	AGG$(TTAGG)_{23}$	1
	---TACAAGTAAgaattc	GGACCGT---GATACC $(A)_{12}$	AGG$(TTAGG)_{25}$	1
	---TACAAGTAAgaattc	GGACCGT---GATACC $(A)_{40}$	AGG$(TTAGG)_{40}$	1

*Nucleotide positions are indicated with the junction of cleavage target site AGG and 3'UTR end, defined as 0. The telomeric repeat-like sequences AGG in the end of inserted copies are underlined and indicated at upstream -394 and -233.
